# Constructing a Prognostic Model for Subtypes of Colorectal Cancer Based on Machine Learning and Immune Infiltration‐Related Genes

**DOI:** 10.1111/jcmm.70437

**Published:** 2025-02-26

**Authors:** Yue Wen, Jing Liao, Chunyan Lu, Lan Huang, Yanling Ma

**Affiliations:** ^1^ Department of Gastrointestinal Surgery, West China Hospital, Sichuan University/West China School of Nursing Sichuan University Chengdu China

**Keywords:** colorectal cancer, core genes, machine learning, prognostic analysis

## Abstract

This study constructed a prognostic model combining machine learning‐based immune infiltration‐related genes in each CRC subtype. We used publicly accessible gene expression data and clinical information on colorectal cancer patients. Integrated bioinformatics analysis was used for the identification of immune‐wise genes. Machine learning algorithms, like LASSO regression and random forest, were utilised to identify the most important genes that may serve as predictors for patient prognosis. Univariate Cox regression, consensus clustering as well as machine learning algorithms were conducted to construct a prognostic risk scoring model. Analysis of functional enrichment, immune infiltration analyses and copy number variations as well as mutational burdens was performed and validated at the single‐cell level. A machine learning‐based model is designed with good predictive power—an area under the receiver operating characteristic curve (AUC‐ROC) of C‐index in cross‐validation. The model also achieved good calibration and discrimination ability to stratify patients into high‐ and low‐risk groups with a statistically significant difference in OS (*p* < 0.05). We have integrated multiple types of gene network features into machine learning systems based on the characteristics of integrating networks with Multi‐Expense Learning algorithms, and we propose a robust approach for predicting CRC molecular subtype patient survival. This model could potentially steer personalised treatment strategies and ameliorate outcomes in patients. Although validation in other cohorts and clinical situations is necessary, it may be useful.

## 1 Introduction

Colorectal cancer (CRC) is a highly prevalent malignant disease which causes great morbidity and mortality worldwide. Although there have been great strides in the diagnosis and treatment of CRC, prognosis can vary widely among patients, which is undoubtedly linked to the heterogeneity of this disease [1, 2, 3]. According to the latest epidemiological data, CRC ranks third in new global cases and second in mortality, with over 1.9 million new cases and 900,000 deaths in 2022. The prognosis of this cancer varies among individuals and is closely related to the heterogeneity of the disease [4, 5]. Therefore, identifying reliable prognostic biomarkers and developing robust predictive models are crucial for improving patient survival rates and optimising personalised treatment strategies. The findings of reliable prognostic biomarkers and developing robust predictive models are vital to increase patient survival and optimise category allocations for personalised therapeutics.

Recent extensive studies have demonstrated that the tumour microenvironment, especially immune infiltration, contributes to CRC progression and prognosis. The immune cells in the tumour microenvironment are known to either encourage or suppress tumour growth, metastasis and response to therapy [6, 7, 8]. As a result, the addition of immune infiltration‐related genes to prognostic models could increase their predictive power and application value.

The emergence of immune‐related biomarkers has further revolutionised the field, as the immune system's role in cancer progression and response to therapy has become increasingly evident. Studies have shown that the tumour microenvironment, particularly immune cell infiltration, is a critical factor in CRC prognosis [9, 10, 11]. The interaction between tumour cells and the immune system is complex, with implications for tumour growth, metastasis and therapeutic response. Despite this, current prognostic models have not fully exploited the potential of immune‐related biomarkers, which may offer additional layers of predictive power.

With the rise in new screening technologies, the complexity and dimension of data are becoming too high for standard statistical testing, and hence it has become important to adopt machine learning tools. Utilising machine learning algorithms, we are able to consider important prognostic genes and build predictive models for patient outcomes [12, 13, 14].

We propose to establish a novel prognostic model for subtypes of CRC according to immune infiltration‐related genes and machine learning. We conducted comprehensive bioinformatics analysis using publicly available gene expression datasets and clinical information of CRC patients to discover potential critical genes. The prognostic model was constructed, and the genes that are most relevant to prognosis were selected using LASSO regression based on gene expression profiling data, combined with machine learning algorithms such as random forest. We further conducted univariate Cox regression, consensus clustering and machine learning algorithms to construct the prognostic risk scoring model. Functional enrichment, immune infiltration analyses and copy number variations were analysed besides the original mutational burdens, which were validated at the single‐cell level.

The aim of this study is to develop a novel prognostic model for CRC subtypes based on immune infiltration‐related genes and machine learning techniques. Identifying key prognostic genes: By integrating bioinformatics analysis of publicly available gene expression datasets and clinical information of CRC patients, key genes that have a significant impact on CRC prognosis are identified to enhance the predictive ability and clinical application value of the model.

## 2 Methods

### 2.1 Data Source

RNA expression profiles and clinical information: The bulk RNA‐seq data as well as the relevant clinicopathological characteristics for lung adenocarcinoma patients were downloaded from TCGA and GEO [15, 16]. Meanwhile, scRNA‐seq of lung adenocarcinoma tissues from GSE146771 was obtained. Colorectal cancer patients were subjected to single‐cell RNA sequencing using SMART‐seq2 and 10× genomic single‐cell 3 ‘library platforms’. Flow cytometry (FACS) was used to enrich myeloid cells with CD45 antibodies and exclude lymphocytes. The raw sequence data can be accessed through the Chinese Genome Sequencing Archive (GSA).

### 2.2 Selection of Core Hub Genes

First, the disease‐related genes’ intersection was identified, and a Venn diagram was generated. Subsequently, the protein–protein interaction network analysis of these intersecting genes was carried out using the STRING website, and the outcomes were imported into Cytoscape for further examination, which led to the identification of six central hub genes. Finally, a functional enrichment analysis of these core hub genes was performed. Functional annotation of the core hub gene set was performed using the Metascape database [17, 18].

### 2.3 Establishment of the Model

Based on the survival status and survival time of patients, the differentially expressed genes related to prognosis are analysed using unsupervised clustering methods to classify patients into several groups for further analysis. Subsequently, a scoring system is constructed using the PCA (principal component analysis) method by selecting components 1 and 2 for this scoring system.

### 2.4 Clinical Functional Assessment

To comprehend the correlation between model scores and clinical characteristics, we examined the association between model scores and individual patient attributes including gender, age, stage, pathology and survival status. Additionally, we confirmed the link between model scores and the survival rates pertaining to various independent clinical features. Univariate and multivariate Cox analyses were conducted to investigate the relationship between model scores and survival rates.

### 2.5 Immune Infiltration Analysis

After grouping the main variables, the data were subjected to corresponding statistical analysis to obtain the distribution of each group within each category. The statistical data were visualised using the ggplot2 package to create overlaid bar charts. Based on the core algorithm of CIBERSORT (CIBERSORT.R script analysis), markers for 22 immune cells provided by the CIBERSORTx website (https://cibersortx.stanford.edu/) were used to calculate the immune infiltration status of the uploaded data. The stromal and immune scores of colorectal cancer patients from TCGA were calculated using the R package—estimate [19, 20].

### 2.6 Machine Learning

Extracting colorectal cancer corresponding TCGA data and matched normal tissue data from GTEx, split at a 1:1:1 ratio into the training set (DatasetA) and three test sets (DatasetB, DatasetC), as well as an internal validation set (DatasetD) randomly sampled from the first three sets. Fifteen machine learning algorithms are employed, including neural network, Lasso regression and naive Bayes, and so forth. For each model, the C‐index is calculated on test sets 1, 2 and 3 and the internal validation set. Models are then ranked based on the average C‐index, AUC area, recall and *F*‐value. LASSO regression achieves feature selection through L1 regularisation, while random forest improves model stability and accuracy by integrating multiple decision trees. We optimise the regularisation parameters of LASSO through cross‐validation and adjust parameters such as the number and depth of trees in the random forest to achieve optimal model performance. Both methods require data preprocessing and rigorous evaluation after model training to ensure the model's generalisation ability and prediction accuracy. To evaluate the stability and generalisation ability of the model, K‐fold cross‐validation was used. The dataset was divided into K subsets, leaving one subset as the test set and the rest as the training set. This process was repeated K times until each subset is used as the test set once. This helps to reduce the variance of model evaluation [21].

### 2.7 Single‐Cell Level Validation

The ‘Seurat’ package in R was utilised for the analysis of single‐cell RNA sequencing (scRNA‐seq) data. Initially, cells with ‘nFeature’ fewer than 200 and ‘percent.mt’ less than 20% were excluded as part of the data quality assessment. Subsequently, single‐cell data from different samples were integrated, and batch effects were mitigated. The ‘LogNormalization’ approach was employed for the unsupervised clustering of cells before visualisation utilising principal component analysis (PCA) and t‐Distributed Stochastic Neighbour Embedding (t‐SNE). The ‘SingleR’ package facilitated the annotation of cell types in each cluster, while the ‘FindAllMarkers’ package was used to detect marker genes exhibiting varying expression levels across distinct cell types. We adopted a conservative approach for handling missing data. In some cases, if the amount of missing data is small, we may use interpolation methods to estimate the missing values. If there is a large amount of missing data, we may choose to exclude these data to avoid introducing bias. The selection of the threshold is based on statistical principles and biological significance. For example, the thresholds for ‘nFeature’ and ‘percentage. Mt’ are determined based on cell biology characteristics and best practices from previous research. These thresholds help balance the integrity of data and the accuracy of analysis [22, 23].

### 2.8 Statistics

All statistical analyses were conducted using the R programming language (Version 4.0.3). Unless specified otherwise, a difference with a *p*‐value of less than 0.05 was deemed statistically significant.

## 3 Results

### 3.1 Comprehensive Overview of Gene Expression Differences Between Normal and Tumour Tissues in CRC

**FIGURE 1:**
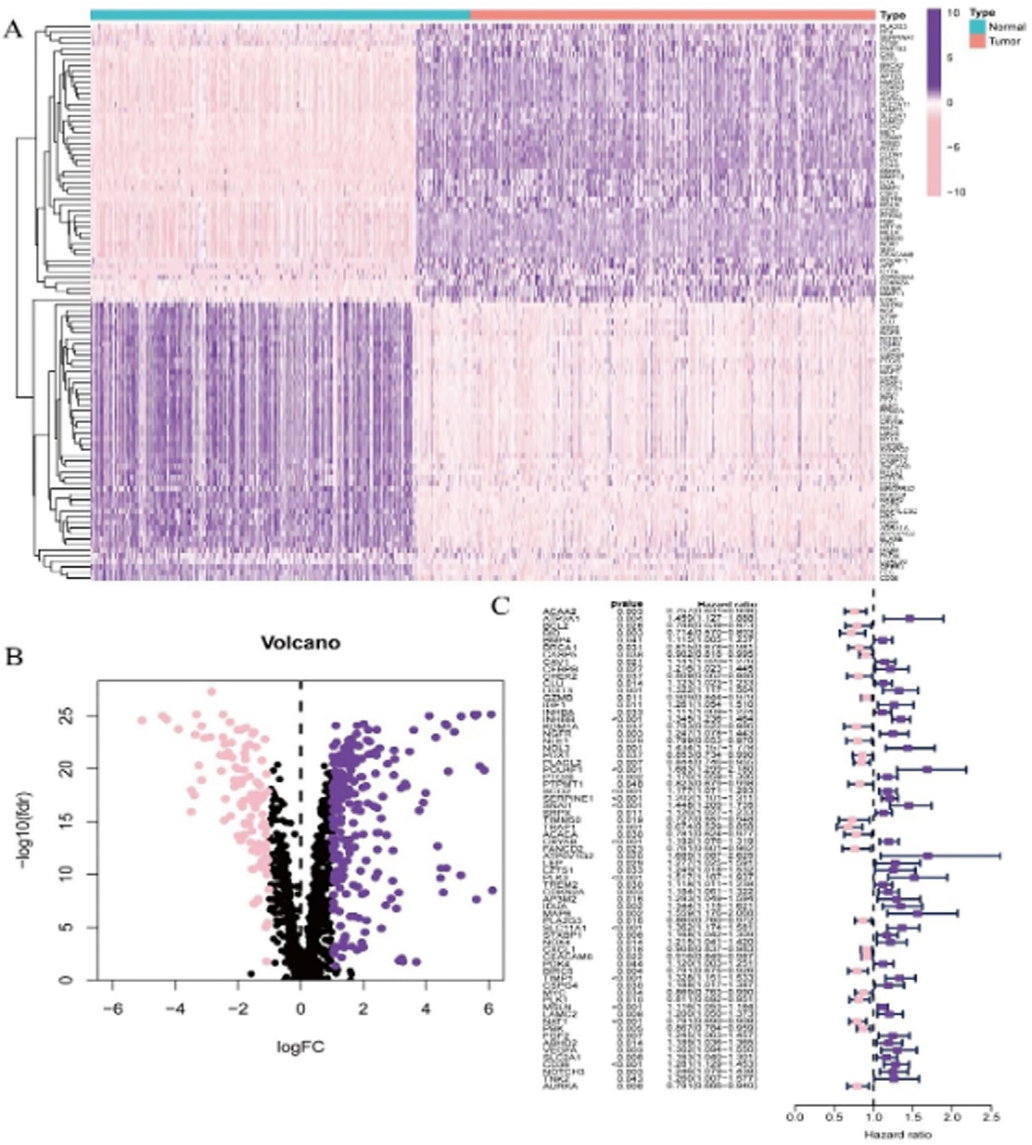
Comprehensive overview of gene expression differences between normal and tumour tissues in CRC. Panel (A) provides a visual representation of the differential expression of genes between normal and tumour samples, highlighting distinct expression patterns and clustering. Panel (B) identifies genes that are significantly upregulated or downregulated in tumour samples compared to normal samples, based on log fold change and *p*‐values. Panel (C) illustrates the impact of core genes on patient survival, with hazard ratios indicating the level of risk associated with each gene's expression.

The heatmap in Figure 1A shows the expression levels of various genes across different samples, with columns representing individual samples and rows representing genes. The volcano plot in Figure 1B displays the differential expression of genes between two conditions, likely normal and tumour tissues. The forest plot in Figure 1C shows the hazard ratios (HR) for various genes, indicating their potential impact on survival.

### 3.2 Consensus Clustering Analysis

**FIGURE 2:**
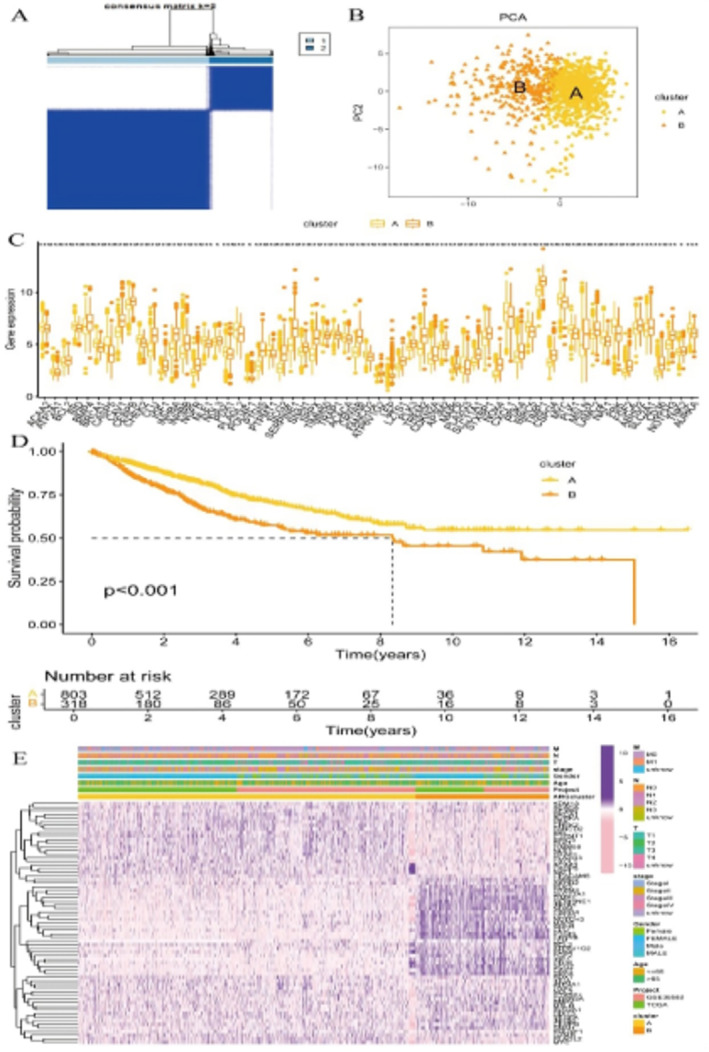
Consensus clustering analysis. Panel (A) shows the stability and robustness of the clustering, indicating two distinct clusters. Panel (B) visualises the separation of the two clusters in a reduced dimensional space, confirming the clustering results. Panel (C) displays the distribution of gene expression levels for each gene across the two clusters, highlighting differences in expression patterns. Panel (D) demonstrates a significant difference in survival probabilities between the two clusters, suggesting that the clustering has prognostic value. Panel (E) provides a detailed view of the gene expression profiles across the samples, with clinical annotations adding context to the data.

Figure 2A shows the consensus clustering matrix for two clusters. The matrix is colour‐coded, with blue indicating high consensus (samples are consistently clustered together) and white indicating low consensus. Two distinct clusters (A and B) are identified, suggesting a clear separation of the samples into these groups. The PCA plot in Figure 2B visualises the separation of the samples into two clusters based on principal components. Figure 2C displays boxplots of gene expression levels for various genes across the two clusters. The Kaplan–Meier survival curve in Figure 2D compares the overall survival between the two clusters. There is a significant difference in survival between the clusters, with Cluster A showing better survival outcomes than Cluster B (*p* < 0.001). The heatmap in Figure 2E displays the gene expression profiles of samples across the two clusters. The heatmap uses a colour gradient from pink (low expression) to purple (high expression) to represent gene expression levels.

### 3.3 Immune Landscape and Biological Pathways Associated With Different Clusters in Colorectal Cancer

**FIGURE 3:**
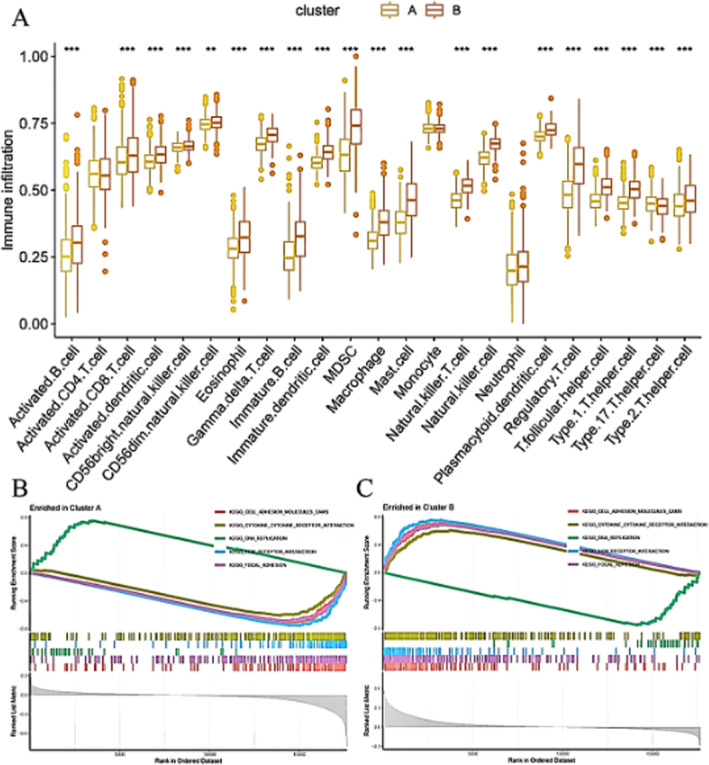
The immune landscape and biological pathways associated with different clusters in colorectal cancer. Panel (A) shows significant differences in the levels of various immune cell types between clusters A and B, suggesting distinct immune microenvironments in the two clusters. Panel (B) indicates that specific gene sets, such as those involved in cell cycle, DNA replication, and mismatch repair, are significantly enriched in Cluster A, suggesting a focus on proliferative and DNA repair processes. Panel (C) indicates that specific gene sets, such as those involved in cell adhesion, JAK–STAT signalling, and cytokine‐cytokine receptor interaction, are significantly enriched in Cluster B, suggesting a focus on cell communication and immune response pathways.

Figure 3A displays boxplots comparing the levels of various immune cell infiltrates between two clusters (A and B) in colorectal cancer. Figure 3B shows the Gene Set Enrichment Analysis (GSEA) for Cluster A. This panel shows the GSEA plot for genes enriched in Cluster A. The y‐axis represents the enrichment score, which indicates the degree to which a gene set is overrepresented at the top or bottom of a ranked list of genes. Figure 3C shows the GSEA plot for genes enriched in Cluster B. Similar to Panel B, the y‐axis represents the enrichment score. The plot highlights gene sets that are significantly enriched in Cluster B, indicating different biological processes or pathways compared to Cluster A.

### 3.4 Comprehensive Analysis of the Prognostic Factors in Colorectal Cancer

**FIGURE 4:**
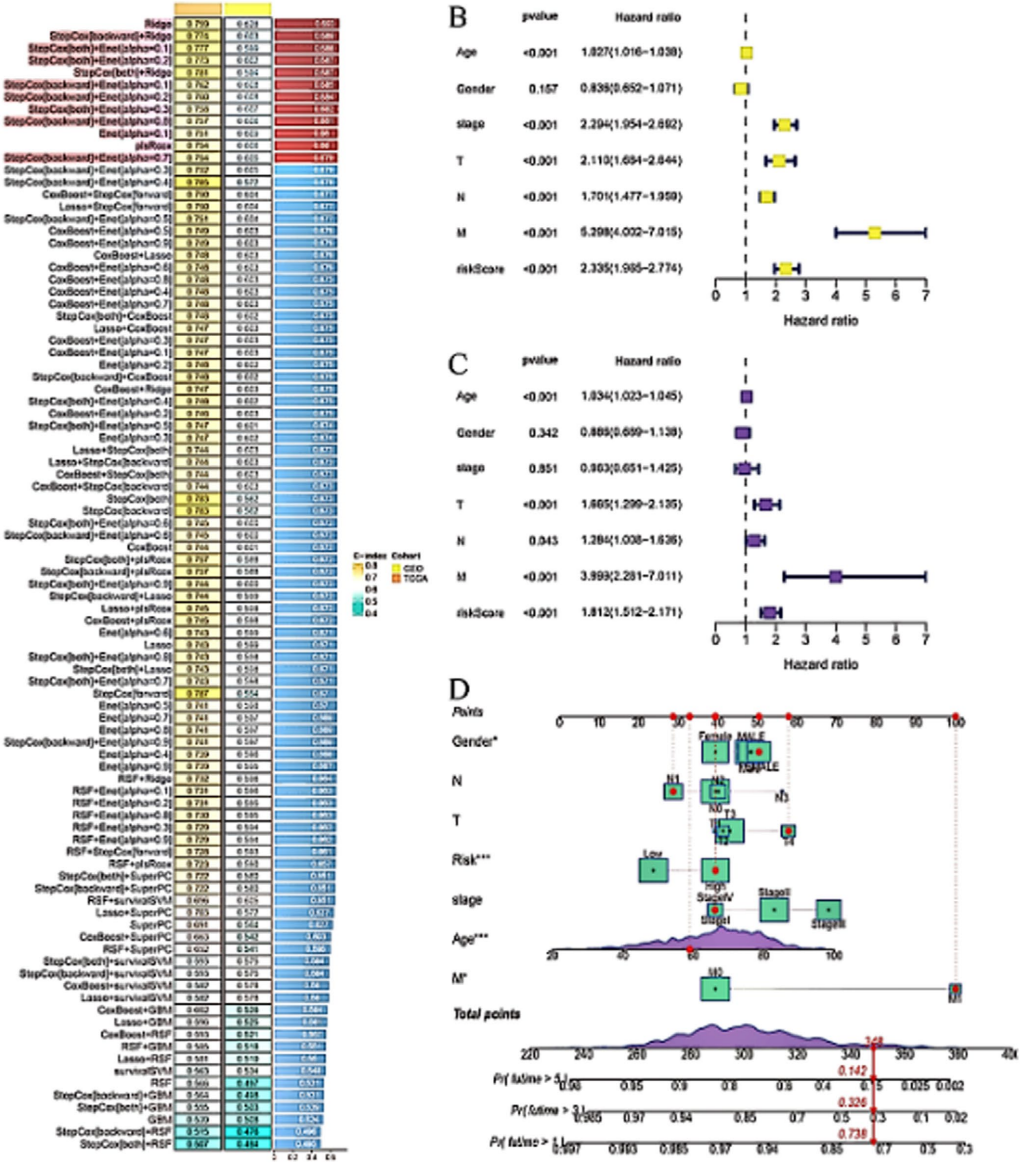
Comprehensive analysis of the prognostic factors in colorectal cancer. Panel (A) highlights the distribution of clinical and molecular features across patient groups. Panel (B, C) shows the results of univariate and multivariate Cox regression analyses, identifying significant prognostic factors. Panel (D) presents a nomogram that integrates these factors to predict patient survival probabilities, offering a practical tool for personalised prognosis and treatment planning.

Figure 4A highlights the distribution of clinical and molecular features across patient groups. Figure 4B,C show the results of univariate and multivariate Cox regression analyses, identifying significant prognostic factors. Figure 4D presents a nomogram that integrates these factors to predict patient survival probabilities, offering a practical tool for personalised prognosis and treatment planning.

### 3.5 Robust Prognostic Performance of the Risk Model in Colorectal Cancer

**FIGURE 5:**
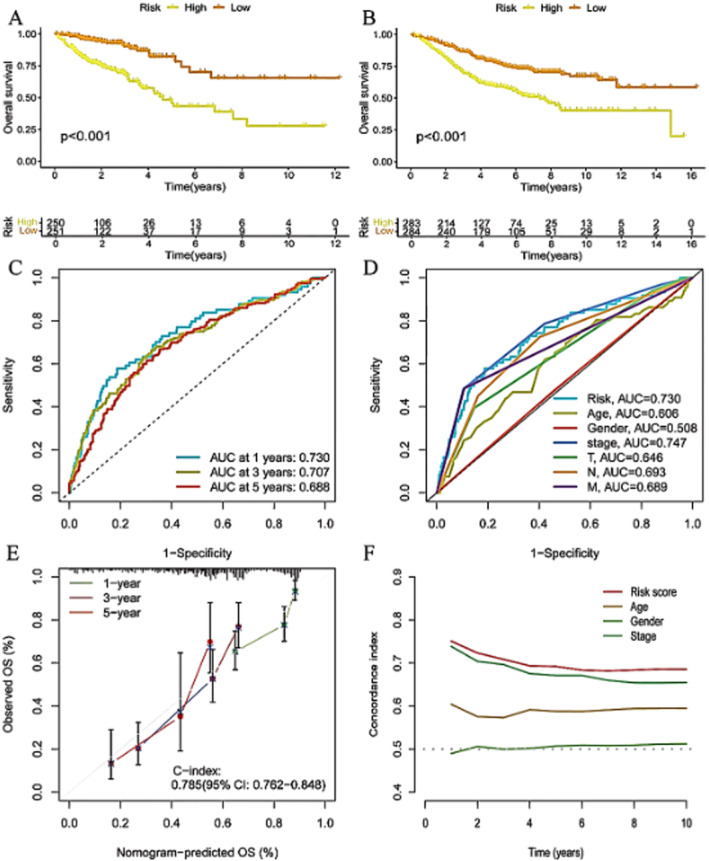
Robust prognostic performance of the risk model in colorectal cancer. Panel (A, B) show significant differences in survival between high‐risk and low‐risk groups in both training and validation cohorts. Panel (C, D) highlight the strong predictive capability of the risk model, with high AUC values. Panel (E) confirms the accuracy of the nomogram through calibration plots. Panel (F) shows the superior prognostic performance of the risk score over time compared to other clinical features.

Figure 5A,B show significant differences in survival between high‐risk and low‐risk groups in both training and validation cohorts. Figure 5C,D highlight the strong predictive capability of the risk model, with high AUC values. Figure 5E confirms the accuracy of the nomogram through calibration plots, and Figure 5F shows the superior prognostic performance of the risk score over time compared to other clinical features. These analyses underscore the potential of the risk model to guide personalised treatment strategies and improve patient outcomes in colorectal cancer.

### 3.6 Analysis of Immune Infiltration in Colon Cancer

**FIGURE 6:**
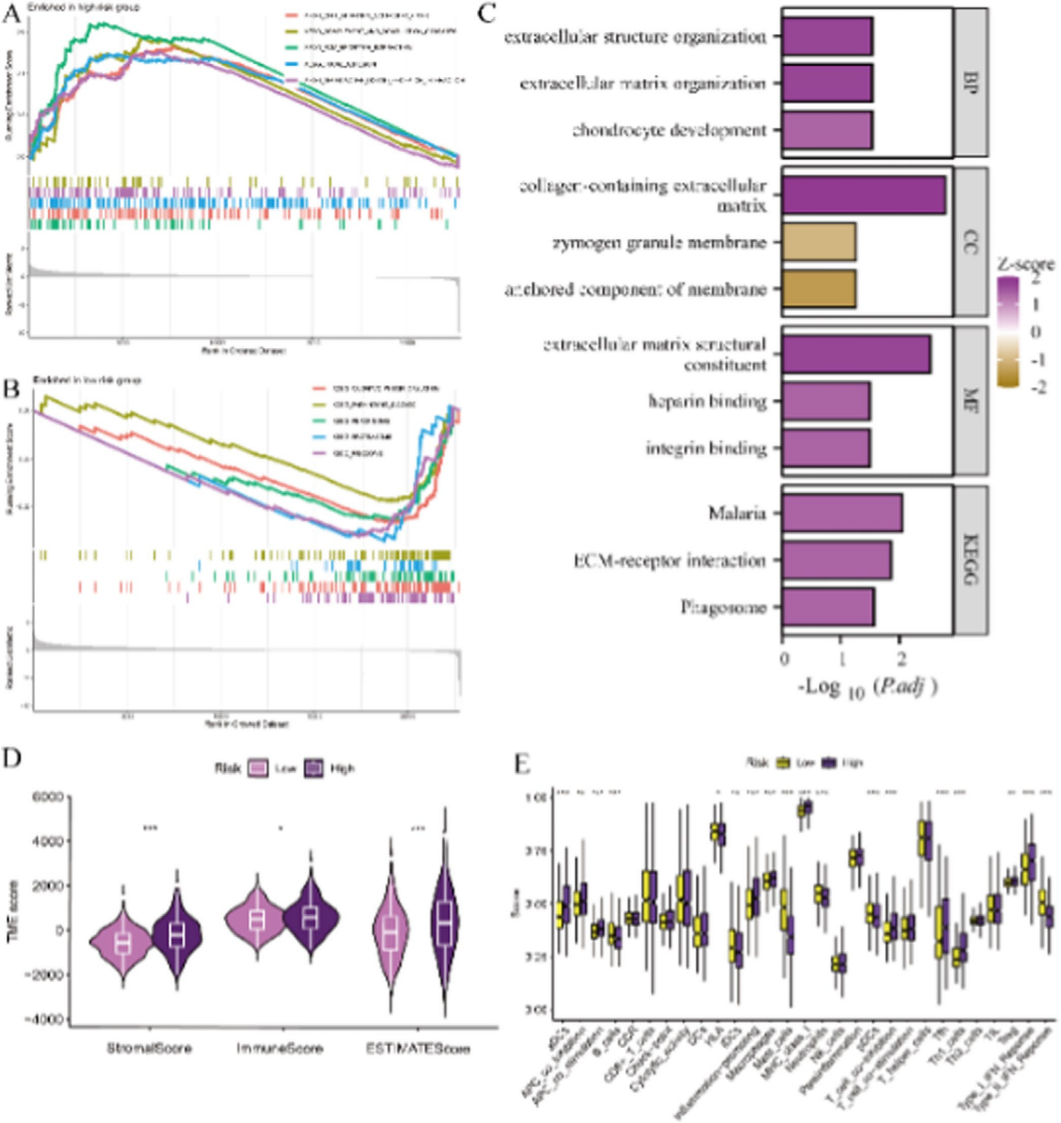
Analysis of immune infiltration in colon cancer. (A, B) Gene Set Enrichment Analysis (GSEA) shows pathways enriched in high‐risk and low‐risk groups, highlighting differences in biological processes and pathways between these groups. (C) Functional Enrichment Analysis identifies significant biological processes (BP), cellular components (CC), molecular functions (MF) and KEGG pathways. Key pathways include extracellular matrix organisation and integrin binding. (D) Violin plots: Compare stromal, immune and ESTIMATE scores between low‐risk and high‐risk groups, indicating significant differences in the tumour microenvironment. (E) Box plots: Display scores for various immune cell types, showing differences in immune cell infiltration between risk groups.

The study utilises various analytical methods to reveal differences in biological characteristics and immune microenvironments between different risk groups. First, Gene Set Enrichment Analysis (GSEA) (Figure 6A) shows that high‐risk groups are enriched in immune‐related and cellular processes, while low‐risk groups are enriched in metabolic and structural pathways. Functional enrichment analysis further indicates that the high‐risk group significantly involves biological processes and functions as extracellular matrix organisation, collagen structure and integrin binding and is associated with KEGG pathways like ECM–receptor interaction and malaria (Figure 6B,C). Comparison of tumour microenvironment scores, including stromal, immune and ESTIMATE scores, reveals significant differences between high‐ and low‐risk groups (Figure 6D). Additionally, immune cell infiltration analysis shows significant differences in the infiltration levels of various immune cell types between risk groups (Figure 6E). These results indicate significant differences in tumour biology and immune characteristics between risk groups, providing important insights for further research on tumour progression mechanisms and personalised therapy.

### 3.7 Comprehensive Analysis of Gene Expression and Interactions

**FIGURE 7:**
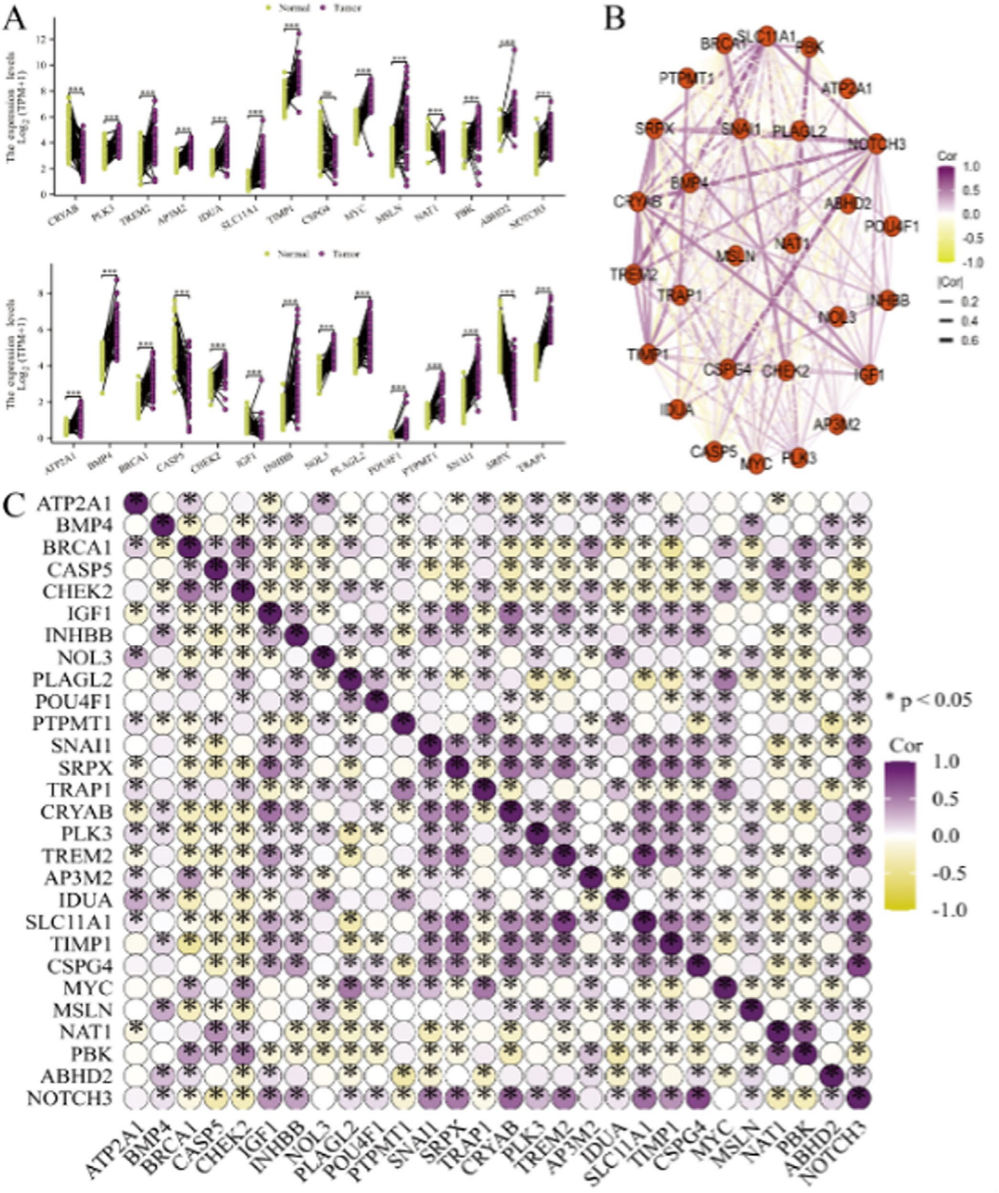
A comprehensive analysis of gene expression and interactions. (A) Expression levels of various genes in normal and tumour tissues. The box plots represent different genes, with significant differences in expression levels between normal and tumour tissues. (B) Gene interaction network: This panel illustrates a network of gene interactions. (C) Correlation matrix: This panel shows a heatmap of the correlation coefficients between pairs of genes.

Figure 7A shows the expression levels of various genes in normal and tumour tissues. The box plots represent different genes, with significant differences in expression levels between normal and tumour tissues. Figure 7B displays the gene interaction network. This panel illustrates a network of gene interactions. The colour gradient from purple to yellow represents the correlation coefficient, where purple indicates a positive correlation and yellow indicates a negative correlation. The network is organised to show the interconnectedness of the genes, highlighting key genes (hubs) with many connections. Figure 7C displays the correlation matrix. This panel shows a heatmap of the correlation coefficients between pairs of genes, represented by the colour gradient, where purple indicates a positive correlation, yellow indicates a negative correlation and white indicates no correlation.

### 3.8 Molecular Interactions Within the Tumour Microenvironment

**FIGURE 8:**
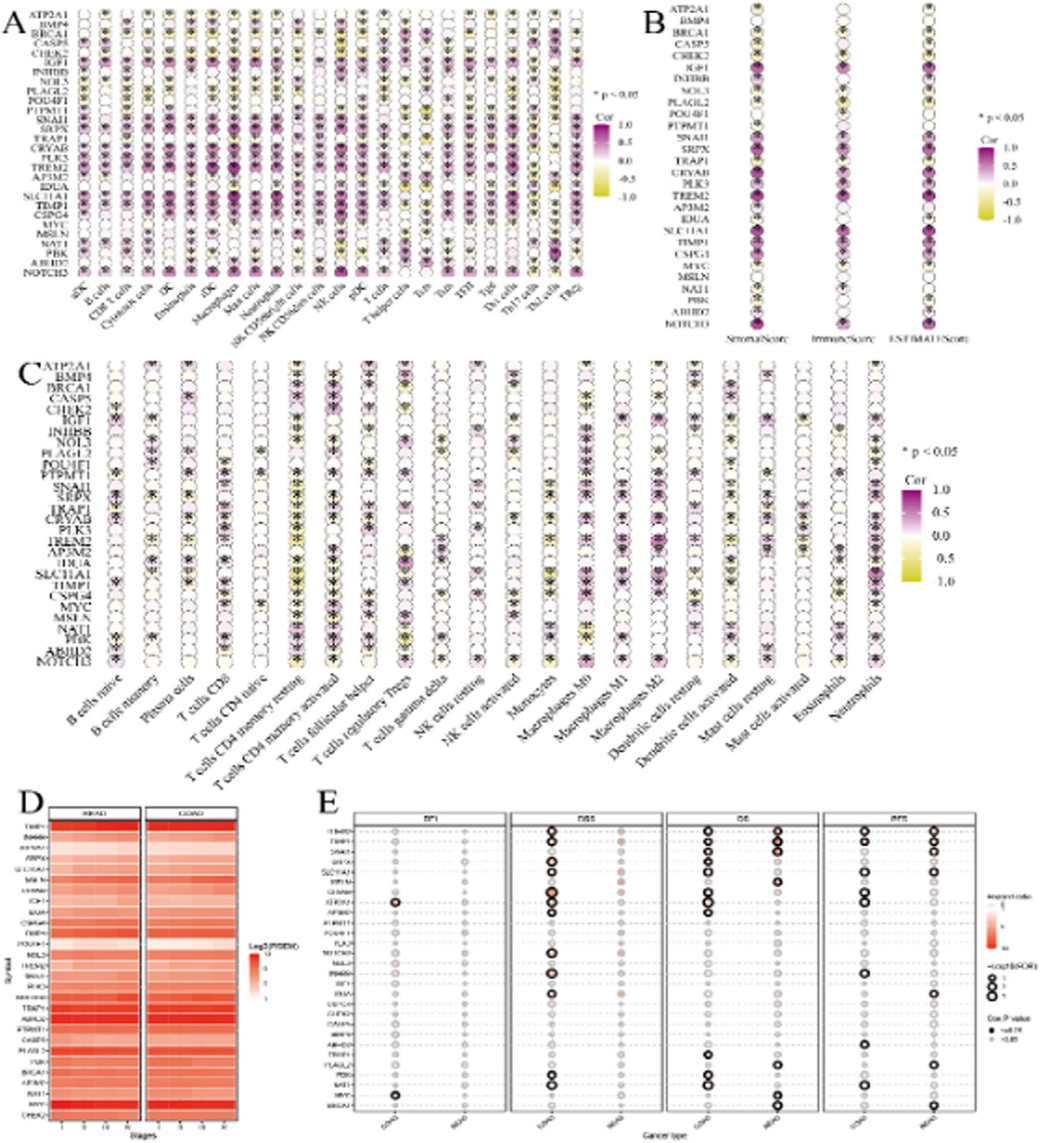
Molecular interactions within the tumour microenvironment. Panel (A, C) provide detailed insights into how the expression levels of various genes correlate with the presence and activity of different immune cell types, highlighting potential interactions and regulatory mechanisms. Panel (B) shows how gene expression correlates with overall stromal and immune scores, which are composite measures of the tumour microenvironment's stromal and immune components. Panel (D) provides a comprehensive view of how the expression levels of various genes vary across different cancer types, highlighting potential biomarkers that may be overexpressed or underexpressed in specific cancers. Panel (E) offers insights into the prognostic significance of gene expression levels across different cancer types and survival metrics, identifying genes that may serve as important prognostic markers for specific cancers.

Figure 8A,C provide detailed insights into how the expression levels of various genes correlate with the presence and activity of different immune cell types, highlighting potential interactions and regulatory mechanisms. Figure 8B shows how gene expression correlates with the overall stromal and immune scores, which are composite measures of the tumour microenvironment's stromal and immune components. The combined analysis across these panels offers a thorough understanding of the relationships between gene expression, immune cell infiltration and the tumour microenvironment, which could inform the development of targeted therapies and prognostic biomarkers. Figure 8D provides a comprehensive view of how the expression levels of various genes vary across different cancer types, highlighting potential biomarkers that may be overexpressed or underexpressed in specific cancers. Figure 8E offers insights into the prognostic significance of gene expression levels across different cancer types and survival metrics, identifying genes that may serve as important prognostic markers for specific cancers.

### 3.9 Comprehensive Analysis of the Mutation Landscape, Its Prognostic Significance and Its Impact on the Tumour Microenvironment

**FIGURE 9:**
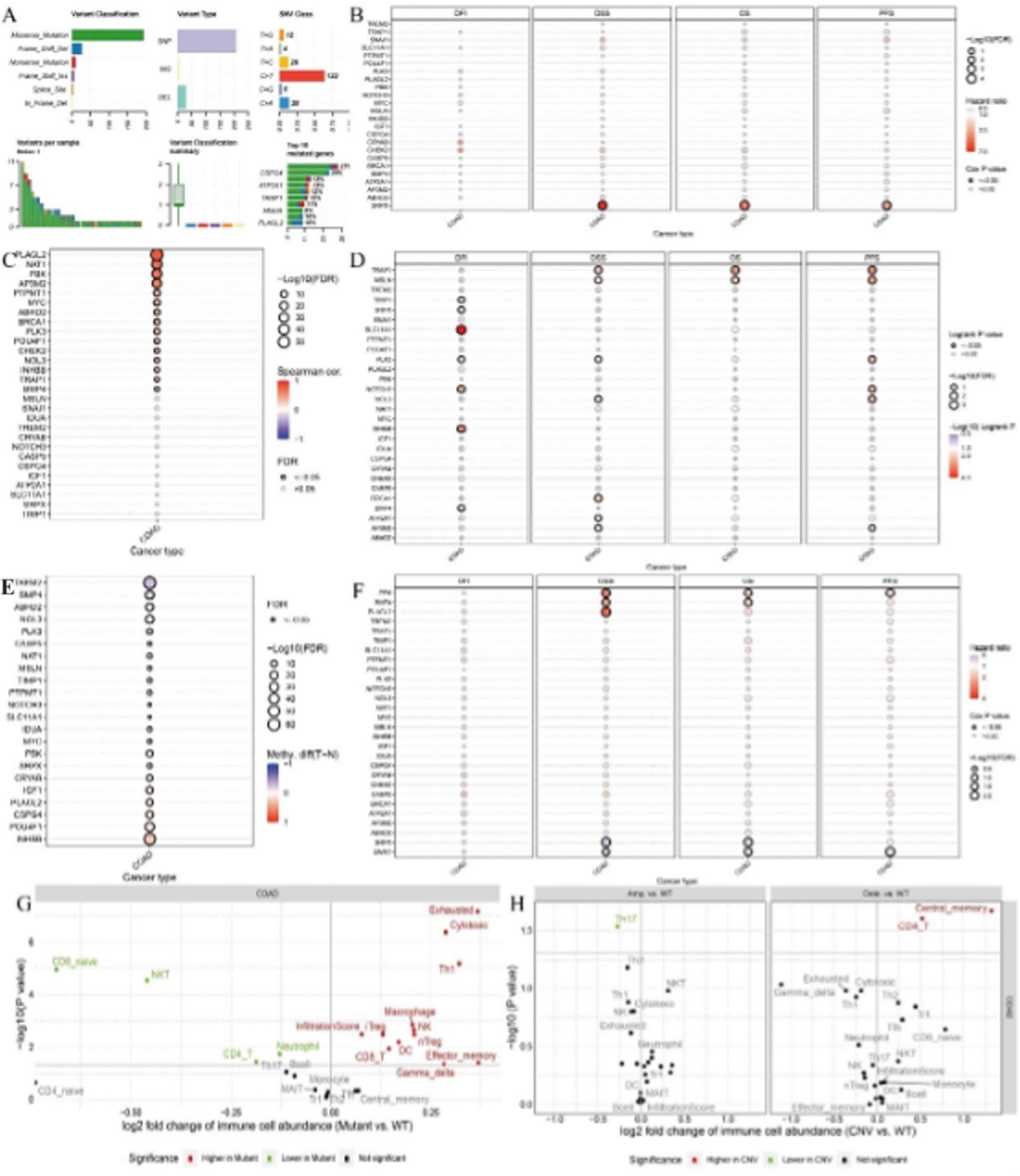
Comprehensive analysis of the mutation landscape, its prognostic significance, and its impact on the tumour microenvironment. Panel (A) provides an overview of the types and frequencies of genetic mutations across samples, highlighting the most commonly mutated genes. Panel (B) shows the association between gene mutations and various survival outcomes across different cancer types. Panel (C–E) show how gene mutations correlate with stromal, immune, and ESTIMATE scores, respectively, across different cancer types. Panel (F) shows the correlation between gene mutations and specific immune cell types. Panel (G, H) provide volcano plots showing changes in immune cell abundance in mutant vs. wild‐type and EN vs. wild‐type, highlighting significant differences.

Figure 9A provides an overview of the types and frequencies of genetic mutations across samples, highlighting the most commonly mutated genes. Figure 9B shows the association between gene mutations and various survival outcomes across different cancer types. Figure 9C–E show how gene mutations correlate with stromal, immune and ESTIMATE scores, respectively, across different cancer types. Figure 9F shows the correlation between gene mutations and specific immune cell types. Figure 9G,H provide volcano plots showing changes in immune cell abundance in mutant vs. wild‐type and EN vs. wild‐type, highlighting significant differences.

### 3.10 Diversity of Cell Types and Their Distribution in Colorectal Cancer Samples

**FIGURE 10:**
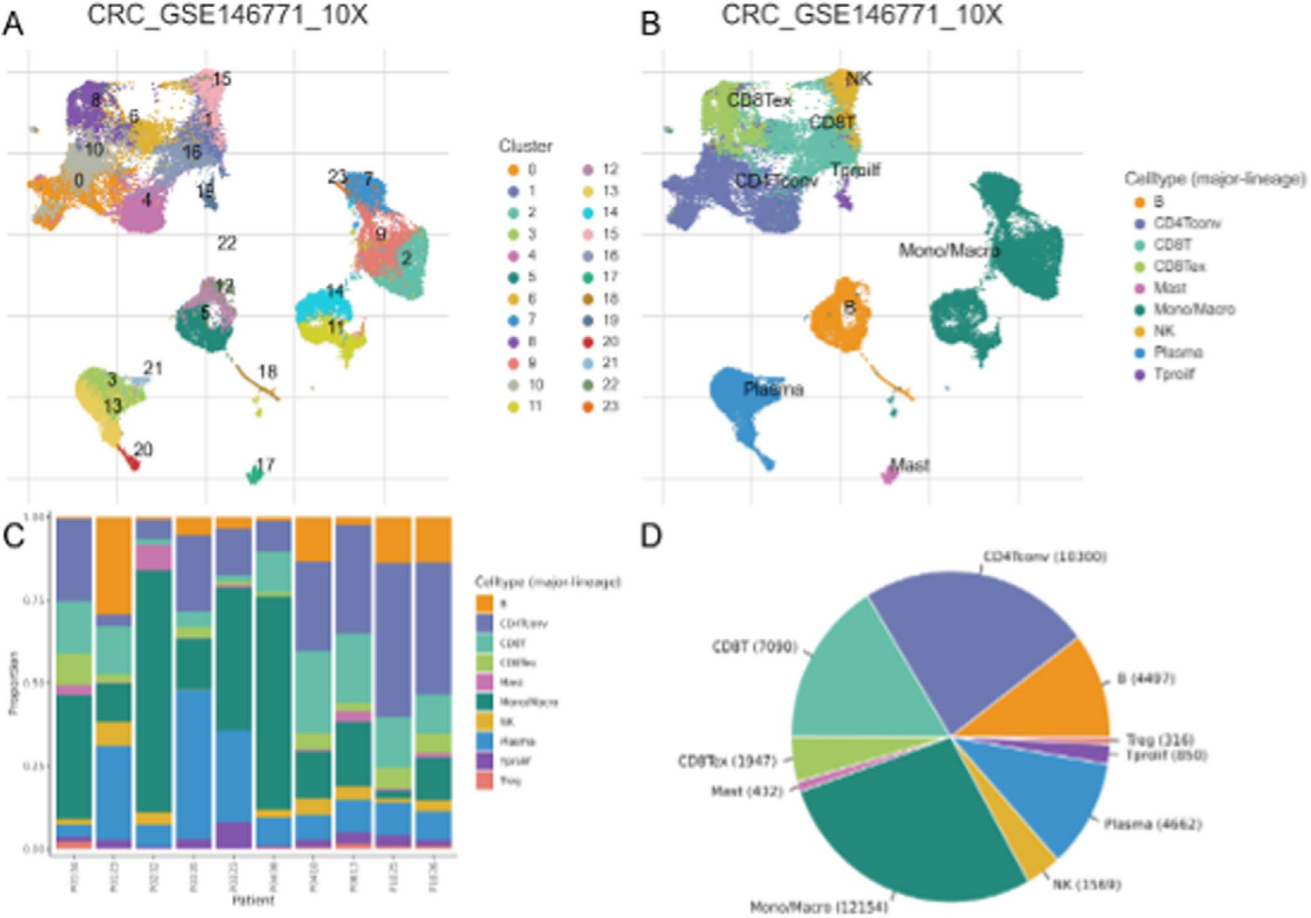
Diversity of cell types and their distribution in colorectal cancer samples. Panel (A) shows the clustering of cells, and Panel (B) shows the distribution of major cell types. Panel (C) shows the proportion of different cell types within each cluster, highlighting the heterogeneity within clusters. Panel (D) provides an overview of the distribution of major cell types across the entire dataset, indicating the relative abundance of each cell type.

Figure 10A,B provides a visual representation of the single‐cell data, with Figure 10A showing the clustering of cells and Figure 10B showing the distribution of major cell types. Figure 10C shows the proportion of different cell types within each cluster, highlighting the heterogeneity within clusters. Figure 10D provides an overview of the distribution of major cell types across the entire dataset, indicating the relative abundance of each cell type.

### 3.11 Expression Patterns of Various Genes in the Single‐Cell RNA Sequencing Dataset

**FIGURE 11:**
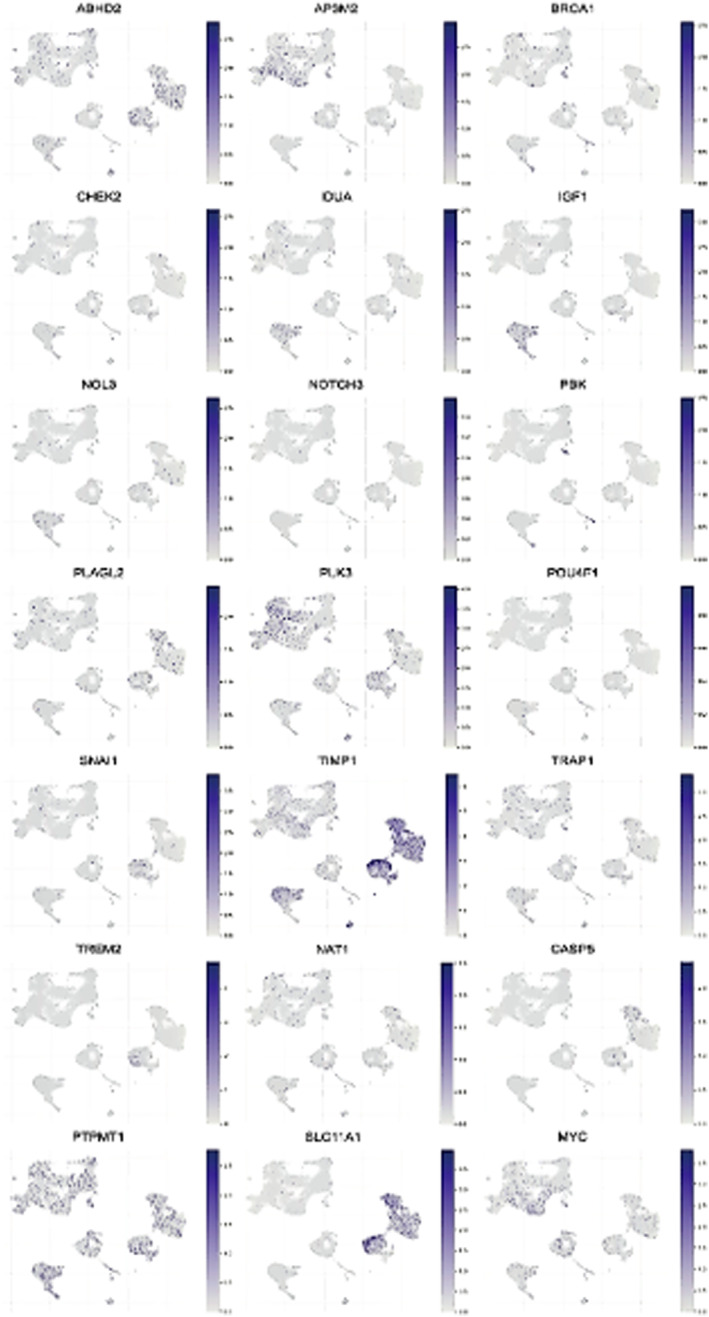
Expression patterns of various genes in the single‐cell RNA sequencing dataset. The UMAP plots show how the expression of each gene is distributed spatially among the different clusters of cells, helping to identify patterns of gene expression in the context of cellular heterogeneity. The expression of genes such as ABHD2, AP3M2, BRCA1, CHEK2, IDUA, IGF1 and NOL3 in cells.

Each UMAP plot provides a visual representation of the expression levels of a specific gene across individual cells. The intensity of the colour indicates the level of expression, with darker shades representing higher expression levels. The UMAP plots show how the expression of each gene is distributed spatially among the different clusters of cells, helping to identify patterns of gene expression in the context of cellular heterogeneity. The expression of genes such as ABHD2, AP3M2, BRCA1, CHEK2, IDUA, IGF1 and NOL3 in cells is shown in Figure 11.

## 4 Discussion

Colorectal cancer (CRC) is a malignancy with high incidence and mortality worldwide. In this study, gene microarray data, clinical information and multiple bioinformatics databases were systematically studied to dissect the relationships as well as molecular mechanisms in CRC [24, 25, 26]. Thirteen core genes were finally determined, which can perform in important biological function areas such as extracellular matrix functions/structure‐affiliated processes and signal transduction, cell growth and protein modification, being relevant to key pathways involved in CRC such as the Wnt signalling. Our findings correlate with previous reports that highlight the significance of Wnt signalling and the TME in CRC initiation and show a crucial part played by ECM. Most of these are core genes involved in tumorigenesis. The key roles of these proteins in extracellular matrix actin, decreased cell proliferation and normal protein modification demonstrate their influence on the tumour microenvironment advocating for cancer progression. In a known driver of CRC development, the study in particular highlights the Wnt signalling pathway. Because of the significant contribution to CRC, malfunctioning activation pathways must lead to non‐controllable cell cycling and eventually create tumours; this is why, it preserves essential functions in CRC.

In the tumour microenvironment of colorectal cancer (CRC) patients, there are multiple types of immune cells that have a significant impact on tumour growth, invasion and response to treatment. The following are the main types and distribution characteristics of immune cells in the tumour microenvironment of CRC patients. CD8 + T cells (cytotoxic T lymphocytes, CTLs) have the ability to directly kill tumour cells by recognising tumour‐specific antigens and releasing cytotoxic molecules such as perforin and granzyme to eliminate tumour cells [27, 28, 29, 30]. Treg cells promote the formation of an immunosuppressive microenvironment by inhibiting the activity of effector T cells, which may inhibit anti‐tumour immune responses and promote tumour immune escape. In TME, tumour‐associated macrophages (TAMs) typically manifest as M2 type, which promote angiogenesis and tumour invasion by secreting Th2 cytokines, while M1 type macrophages exert pro‐inflammatory and anti‐tumour effects.

The ABHD2 (Abhydrolase domain containing 2) gene codes for an enzyme of the α/β‐hydrolase superfamily which is known to play a major role in lipid metabolism, especially fatty acid metabolism. Recent studies described that the ABHD2 gene may contribute to the process of multiple tumour development, invasion and metastasis. In addition, the level of ABHD2 gene expression is altered in cancers, and its normal function has been implicated in tumour cell proliferation and survival. Given the role of ABHD2 in fatty acid metabolism, we speculate that this gene might play a valuable part in tumour growth and progression through metabolic reprogramming of tumour cells. ABHD2 regulates extracellular matrix and cell–cell interactions, influencing tumour invasion and metastasis [31, 32].

Adaptor Protein 3 Complex Mu Subunit 2 (AP3M2) is a subunit of the AP‐3 complex, which functions in protein sorting, vesicle transport and cargo‐specific concentrations involved in receptor‐mediated endocytosis. The AP‐3 complex is responsible for the transport and sorting of a subset of proteins within cells, most likely functioning to mediate protein transfer from the Golgi apparatus to lysosomes (1), as well as between ERGIC (−endoplasmic reticulum–Golgi intermediate compartment) and other organelles. It may regulate tumour cell growth and differentiation by participating in protein sorting during the transport of proteins from the Golgi to lysosomes. Its functional disruption can result in cellular stress responses, endoplasmic reticular stress and activated unfolded protein response with the subsequent release of apoptotic signals leading to death towards managing intracellular environmental stability that may have impaired the survival of tumour cells. Based on these results, the expression profile of AP3M2 changed as HCT‐116 cells become more aggressive in vitro—suggesting that AP3M2 may be involved in extracellular matrix remodelling and cell adhesion molecule regulation—for colon cancer invasion and metastasis [33, 34, 35, 36, 37].

Breast cancer type 1 susceptibility protein (BRCA1) gene produces a breast and ovarian‐cancer‐specific tumour suppressor that plays roles in the control of the cell cycle checkpoint and maintenance of chromosomal stability. Loss of BRCA1 has been shown to be responsible for numerous tumour types, and it is linked not only with the initiation but also the progression of tumours, especially in breast and ovarian cancers [38, 39, 40].

The model integrates immune infiltration data, which is relatively novel in CRC prognostic models; second, advanced machine learning techniques such as LASSO regression and random forest have been adopted, which have shown advantages in improving the predictive ability of the model; Finally, attention was paid to the generalisation ability of the model, which has not been fully emphasised in many existing studies [41, 42].

Additionally, immune infiltration analysis revealed a hint of the possibility in understanding how immunotherapy can be effective to treat CRC. This study implies that the tumour microenvironment of CRC patients has features favouring immune‐based therapeutic strategies and is in accordance with the current trends for personalised to targeted immunotherapeutic research focusing on efficient ways how we can use our own immunity against cancer. Moreover, the prognostic‐related analysis and immune infiltration analysis further confirmed that core genes might be related to CRC prognosis as well; meanwhile, these were screened for being critical in immune response. It has been proved that the CDKN2A and TIMP1 genes were two of them which are closely related to CRC prognosis. Moreover, the findings of immune infiltration patterns associated with these signatures indicate that immunotherapy also harbours important value for treating CRC in accordance with current attention on immunotherapies. Further exploration of personalised treatment plans based on patient‐specific genetic background, tumour molecular characteristics and immune microenvironment is needed to improve treatment efficacy and reduce side effects. In the future, research will be conducted on how to enhance the effectiveness of immunotherapy by regulating immune cell infiltration in the tumour microenvironment, including the development of new immune checkpoint inhibitors and cell therapies.

### 4.1 Limitations

The performance of machine learning models largely depends on the quality and representativeness of the training data. If there is bias or incompleteness in the dataset, the model may not be able to accurately capture all relevant features of CRC, which affects the predictive ability of the model. Although machine learning techniques such as LASSO regression and random forest perform well in predictive performance, these models are often considered “black box” models, and their internal decision‐making processes are difficult to explain. This limits the application of the model in clinical decision‐making, as doctors and researchers may need to understand the predictive basis of the model. The prognostic model developed in this study may perform well on specific datasets, but its generalisation ability to other populations or clinical environments has not been fully validated. The model may face the risk of overfitting, where it performs well on training data but deteriorates on new, unseen data.

## 5 Conclusions

This model could potentially steer personalised treatment strategies and ameliorate outcomes in patients. Although validation in other cohorts and clinical situations is necessary, it may be useful.

## Author Contributions

**Yue Wen:** conceptualization (equal), data curation (equal), writing – original draft (equal). **Jing Liao:** formal analysis (equal), investigation (equal). **Chunyan Lu:** formal analysis (equal), validation (equal). **Lan Huang:** data curation (equal), methodology (equal). **Yanling Ma:** project administration (equal), supervision (equal), writing – review and editing (equal).

## Conflicts of Interest

The authors declare no conflicts of interest.

## Data Availability

The datasets analyzed in this study are publicly available, with details provided in the 2. Methods section. Further information is available from the corresponding author.
